# Comparison of optical quality and distinct macular thickness in femtosecond laser-assisted versus phacoemulsification cataract surgery

**DOI:** 10.1186/s12886-020-1319-3

**Published:** 2020-02-01

**Authors:** Yong Wang, Jinling Zhang, Miaomiao Qin, Jianguo Miao, Wei Chen, Yemeng Huang, Jian Wu, Yu Guan, Huaijin Guan

**Affiliations:** 1 Department of Ophthalmology, Affiliated Hospital of Nantong University, 20 Xisi Road, Nantong, Jiangsu China; 2 Nantong University, Nantong, Jiangsu China

**Keywords:** Optical quality, Macular thickness, Femtosecond laser-assisted cataract surgery (FLACS), Phacoemulsification cataract surgery (PCS), Dysfunctional lens index (DLI), Point spread function (PSF), Modulation transfer function (MTF)

## Abstract

**Background:**

Optical quality and macular thickness changing optical quality is rarely reported after femtosecond laser-assisted cataract surgery (FLACS). In current research, we evaluated optical quality recovery and distinct macular thickness changes after FLACS and phacoemulsification cataract surgery (PCS).

**Methods:**

A total of 100 cataract patients (100 eyes) were included (50 eyes for the FLACS group and 50 eyes for the PCS group). Modulation transfer function (MTF), point spread function (PSF) and dysfunctional lens index (DLI) were measured by a ray-tracing aberrometer (iTrace). Uncorrected distance visual acuity (UDVA) and corrected distance visual acuity (CDVA) were also assessed pre-operation,1 week and 1 month after surgery. The MTF values at spatial frequencies of 5, 10, 15, 20, 25 and 30 cycles/degree (c/d) were selected. We used optical coherence tomography (OCT) to assess the macular thickness of different regions pre-operatively and1month after the surgery.

**Results:**

In PCS group, we found the statistically significant differences between pre-operation and post-operation in DLI (*p* < 0.0001), PSF (strehl ratio, SR) (*p =* 0.027) and MTF (*p* = 0.028), but not intraocular pressure (IOP) (*p* = 0.857). The differences between pre-operation and post-operation for DLI (*p* = 0.031), SR (*p* = 0.01) and IOP (*p* = 0.03), but not MTF (*p* = 0.128) were also found in FLACS group. The differences were statistically significant when the spatial frequencies were at 5, 10 and 25 (*p* = 0.013, 0.031 and 0.048) between pre-operation and post-operation in PCS group but not FLACS group at 1 month. In PCS group, we found the differences between pre-operation and post-operation in nasal inter macular ring thickness (NIMRT) (*p* = 0.03), foveal volume (FV) (*p* = 0.034) and average retinal thickness (ART) (*p* = 0.025) but not FLACS group at 1 month.

**Conclusion:**

FLACS is safe that did not cause significant increase of macular thickness in current study. However, it also cannot produce better optical quality. In contrast, PCS can produce macular thickness changes, but better optical quality recovery. The slightly retinal change may not affect optical quality.

## Background

Femtosecond laser-assisted cataract surgery (FLACS) has gained popularity in recent years. It is the new technology suggesting potential improvements in clinical and safety outcomes over conventional phacoemulsification cataract surgery (PCS) [1, 2]. Many studies have highlighted that little clinically meaningful benefit exists from femtosecond laser pretreatment to cataract surgery [3, 4]. Even conventional PCS had a higher rate of cystoid macular edema (CME) than FLACS, research showed FLACS did not yield better visual or refractive outcomes than PCS [4, 5]. Our previously study have illustrated that FLACS did not result in macular thickness changes in cataract patients with myopia [6]. However, weather the impact of FLACS on optical quality and macular thickness changing optical quality is rarely reported.

Vision can be assessed in terms of visual acuity and optical quality. The optical quality refers to the evaluation of the optical beam from the cornea to the retina. The global point spread function analysis are objective measurements of this optical quality [7]. In order to correct vision after cataract surgery, it is important to evaluate not only visual acuity but also optical quality. More and more researches focus on the relationship between intraocular lens (IOL) implantation and optical quality recovery [8, 9]. However, few studies have investigated the changes in or influences on optical quality if the retina itself is not normal after cataract surgery.

Researcher analyzed the effects of retinal change on optical quality in central serous chorioretinopathy patients and found retinal change affected optical quality [10]. Retinal change especially macular thickness might be a sub-threshold retinal injury after FLACS and warrants further study [11]. A large comparative cohort study identified a higher rate of CME after FLACS [4]. However, other studies have shown that FLACS does not appear to increase macular thickness or CME more than PCS [3, 12–14]. Our previously study have shown that FLACS is safe for cataract patients with myopia that did not change the macular thickness [6]. However, we did not focus on the relationship between optical quality and macular thickness changes in the research.

In recent years, increasing scholars want to provide good optical quality for patients after cataract surgery. It is important to assess optical quality after the surgery. Modulation transfer function (MTF) is an objective method to evaluate imaging quality for human optical systems [15]. We also used strehl ratio (SR) to evaluate optical quality that is ideal PSF to aberrated PSF. It is defined as the ratio between the MTF area of the eye and the diffraction-limited MTF area [16]. Therefore, we assessed optical quality with MTF and SR in the study. Dysfunctional lens index (DLI) is a term coined to describe the natural aging changes in the lens as a novel surgery decision-maker [17, 18]. In current study, we evaluated compare the optical quality outcomes and distinct macular thickness changes in FLACS and PCS group. In addition, we also analyzed the MTF values at spatial frequencies of 5, 10, 15, 20, 25 and 30 cycles/degree.

## Methods

This observational prospective cohort study reviewed 100 patient records of FLACS and PCS cases performed by a single surgeon at Affiliated Hospital of Nantong University from January 2018 to September 2018. The study approved by the institutional ethics committee of Affiliated Hospital of Nantong University and was performed according to the tenets of the Declaration of Helsinki. All patients were willing to volunteer for the research and signed a written informed consent. We excluded the patients with previous ocular surgery, trauma and known macular alteration and all patients were given a complete ophthalmologic evaluation before surgery as our previously study. Uncorrected distance visual acuity (UDVA) and corrected distance visual acuity (CDVA) with subjective refraction performed by an optometrist using an LCD visual acuity chart preoperatively and 1 month postoperatively. Emery-Little classification was used to define the nuclear hardness. The Lens Opacities Classification System III (LOCS III) was used to define the lens opacities. The basic demographic of the study participant were listed in the Additional file 1: Table S1.All FLACS were performed by the same surgeon (H.J.G.) as our previously described [6]. All PCS surgeries also were performed by the same surgeon (H.J.G.). Tobramycin and Dexamethasone Eye Drops (Tobradex, Alcon) were used four times daily after surgery until day 14. Tobramycin and Dexamethasone Eye Ointment (Tobradex, Alcon) was used one time a day after surgery for 30 days. Pranoprofen eye drops (Pranopulin, Senju Pharmaceutical Co., Ltd.) was used three times a day after surgery for 30 days.

### Ray-tracing Aberrometry

A wavefront aberrometry scan was performed with a ray-tracing aberrometer (iTrace, Tracey Technologies, Houston, TX) preoperatively and at the final 1 month follow-up as previously described [19]. Using the iTrace, we measured the optical quality parameters (MTF and SR) of the eye and DLI. For the MTF values measured at spatial frequencies, we selected 5, 10, 15, 20, 25 and 30 cycles/degree (c/d). Pupils were dilated with a 6-mm size before measurement.

### OCT measurements

OCT measurements (Cirrus HD-OCT 4000; Carl Zeiss Meditec, Dublin, CA) were performed 1 day before surgery and post-operation at 1 month as our previously described [6].

### Statistical analysis

A SPSS 18.0 software (SPSS Inc., Chicago, Illinois) was performed for statistical analyses. Data are expressed as the mean and standard deviation. One-way ANOVA was performed to make comparisons between multiple subgroups for different macular region thickness, MTF, SR and DLI between FLACS group and PCS group in pre-operation or post-operation. A *p* value less than 0.05 was considered statistically significant.

## Results

The FLACS group comprised of 50 eyes of 50 patients with the mean age of 56.66 ± 5.68 years. The PCS group comprised of 50 eyes of 50 patients with the mean age of 61.33 ± 7.52 years (Table 1). Preoperative intraocular pressure (IOP), AL, DLI, SR, MTF, UDVA, CDVA and nuclear hardness showed no statistically significant between two groups. We also found FLACS group produce less cumulative dissipated energy (CDE) but not ultrasound time when compared with PCS group (Table 1). 
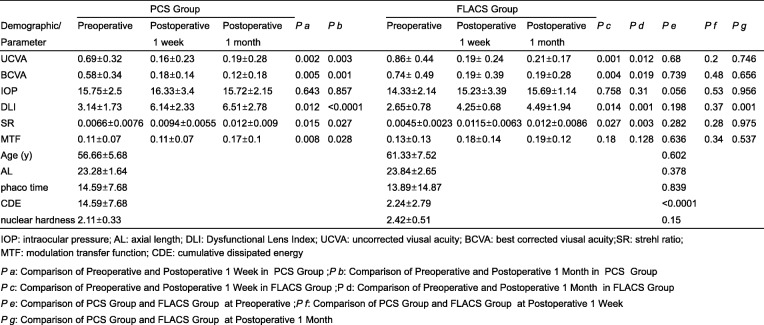

**Table 1:** Comparison of PCS Group and FLACS Group at Preoperative, Postoperative, 1 week and Postoperative 1 month

Demographic/Parameter	PCS Group			*P a*	*P b*	FLACS Group			*P c*	*P d*	*P e*	*P f*	*P g*
	Preoperative	Postoperative	Postoperative			Preoperative	Postoperative	Postoperative					
		1 week	1 month				1 week	1 month					
UCVA	0.69±0.32	0.16±0.23	0.19±0.28	0.002	0.003	0.86± 0.44	0.19± 0.24	0.21±0.17	0.001	0.012	0.68	0.2	0.746
BCVA	0.58±0.34	0.18±0.14	0.12±0.18	0.005	0.001	0.74± 0.49	0.19± 0.39	0.19±0.28	0.004	0.019	0.739	0.48	0.656
IOP	15.75±2.5	16.33±3.4	15.72±2.15	0.643	0.857	14.33±2.14	15.23±3.39	15.69±1.14	0.758	0.031	0.056	0.53	0.956
DLI	3.14±1.73	6.14±2.33	6.51±2.78	0.012	<0.0001	2.65±0.78	4.25±0.68	4.49±1.94	0.014	0.001	0.198	0.37	0.015
SR	0.0066±0.0076	0.0094±0.0055	0.012±0.009	0.015	0.027	0.0045±0.0023	0.0115±0.0063	0.012±0.0086	0.027	0.003	0.282	0.28	0.975
MTF	0.11±0.07	0.11±0.07	0.17±0.1	0.008	0.028	0.13±0.13	0.18±0.14	0.19±0.12	0.018	0.128	0.636	0.34	0.537
Age (y)	56.66±5.68					61.33±7.52					0.14		
AL	23.28±1.64					23.84±2.65					0.378		
phaco time	14.59±7.68					13.89±14.87					0.839		
CDE	14.59±7.68					2.24±2.79					<0.0001		
nuclear hardness	2.11±0.33					2.42±0.51					0.15		

*IOP* intraocular pressure, *AL* axial length, *DLI* Dysfunctional Lens Index, *UCVA* uncorrected viusal acuity, *BCVA* best corrected viusal acuity, *SR* strehl ratio, *MTF* modulation transfer function, *CDE* cumulative dissipated energy

*P a*: Comparison of Preoperative and Postoperative 1 Week in PCS Group; *P b*: Comparison of Preoperative and Postoperative 1 Month in PCS Group

*P c*: Comparison of Preoperative and Postoperative 1 Week in FLACS Group; *P d*: Comparison of Preoperative and Postoperative 1 Month in FLACS Group

*P e*: Comparison of PCS Group and FLACS Group at Preoperative; *P f*: Comparison of PCS Group and FLACS Group at Postoperative 1 Week

*P g*: Comparison of PCS Group and FLACS Group at Postoperative 1 Month

The postoperative IOP (*p* = 0.53; *p* = 0.956), SR (*p* = 0.53; *p* = 0.975) and MTF (*p* = 0.28; *p* = 0.537) no statistically significant between two groups, except DLI (*p* = 0.015) at 1 month. For UDVA and CDVA, we detected no significance between the two groups postoperatively at 1 week or 1 month. In PCS group, there are different between pre-operation and post-operation at 1 week and 1 month in DLI (*p* = 0.012; *p* < 0.0001), SR (*p* = 0.015; *p* = 0.027) and MTF (*p* = 0.008; *p* = 0.028), but not IOP (*p* = 0.643; *p* = 0.857) (Table 1). In FLACS group, we found there are different between pre-operation and post-operation at 1 week or 1 month in DLI (*p* = 0.014; *p* = 0.001), SR (*p* = 0.027; *p* = 0.003), but not MTF (*p* = 0.18; *p* = 0.128) and IOP (*p* = 0.758; *p* = 0. 31) (Table 1).

**Fig. 1:**
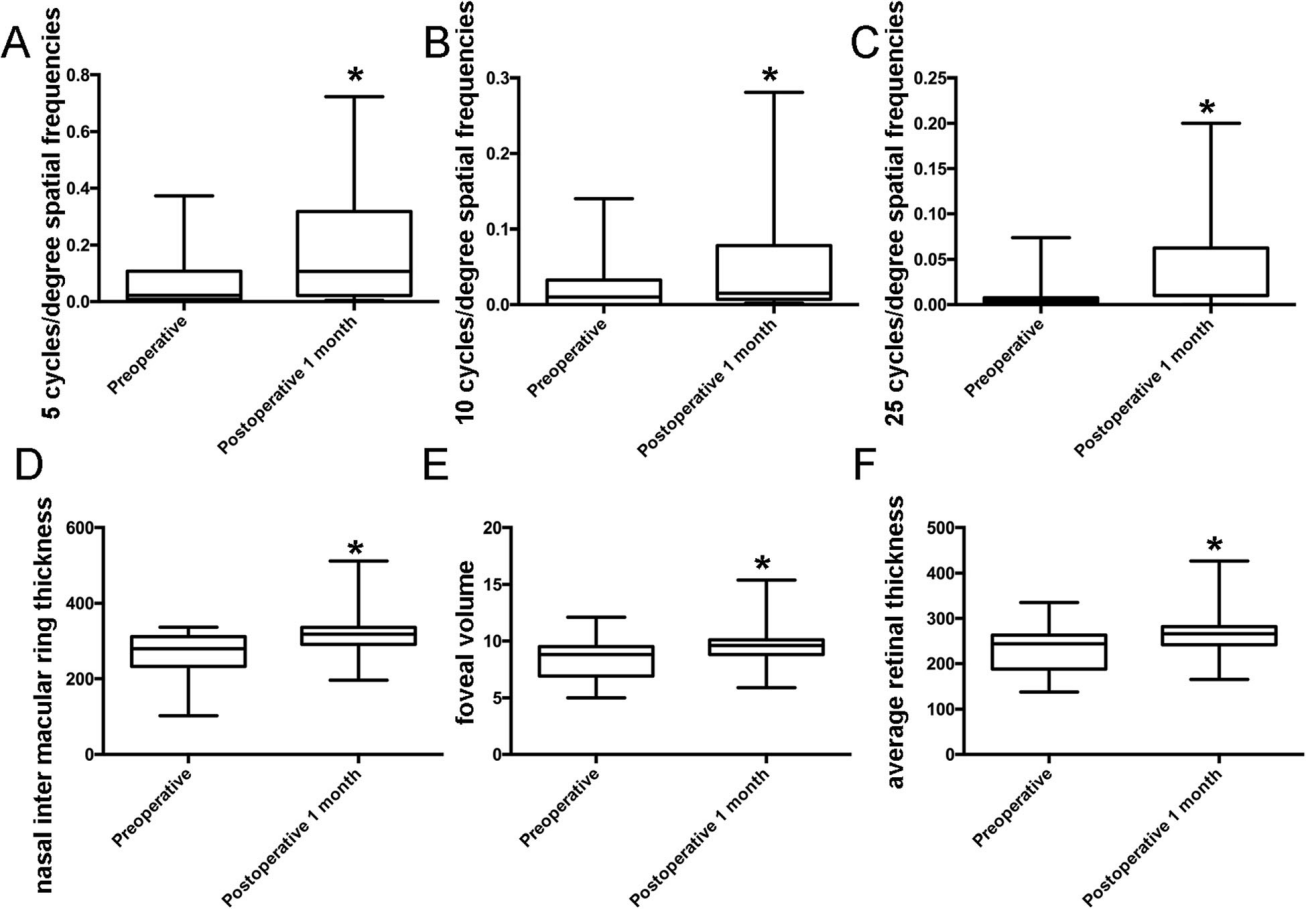
Comparison difference spatial frequencies MTF and retinal thickness values of preoperative and postoperative 1 month for PCS group. Comparison of 5(**a**),10(**b**) and 25 cpd (**c**) between preoperative and postoperative 1 month. Comparison of NIMRT(**d**), FV(**e**) and ART (**f**)between preoperative and postoperative 1 month, *: *p* < 0.05

MTF values at 5, 10, 15, 20, 25 and 30 cycles/degree (c/d) spatial frequencies were obtained pre-operation,1 week and 1 month post-operation by iTrace. The comparison of MTF values at the same spatial frequencies showed rarely statistically significant between two groups pre-operation,1 week and 1 month post-operation (Table 2). In PCS group, we did not find any statistically significant at 1 week. We also did not find any statistically significant at 1 month for 15, 20 and 30 cycles/degree (c/d) spatial frequencies, except 5, 10 and 25 cpd (*p* = 0.013,0.031 and 0.048) (Table 2, Fig. 1a-c). However, there are not any statistically significant between pre-operation, 1 week and 1 month post-operation in FLACS group (Table 2). 
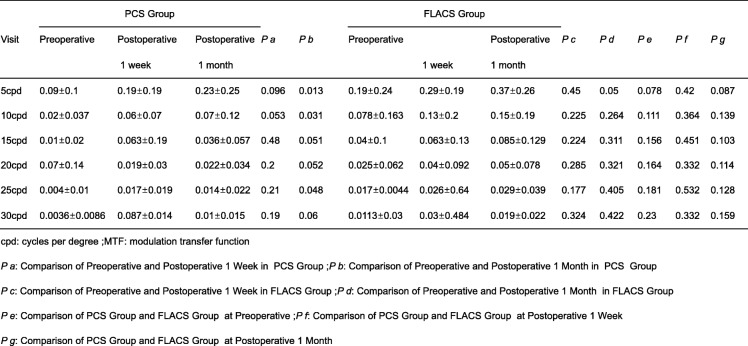

**Table 2:** Preoperative and Postoperative difference spatial frequencies MTF comparison for PCS group and FLACS group

Visit	PCS Group			*P a*	*P b*	FLACS Group			*P c*	*P d*	*P e*	*P f*	*P g*
	Preoperative	Postoperative	Postoperative			Preoperative		Postoperative					
		1 week	1 month				1 week	1 month					
5cpd	0.09±0.1	0.19±0.19	0.23±0.25	0.096	0.013	0.19±0.24	0.29±0.19	0.37±0.26	0.45	0.05	0.078	0.42	0.087
10cpd	0.02±0.037	0.06±0.07	0.07±0.12	0.053	0.031	0.078±0.163	0.13±0.2	0.15±0.19	0.225	0.264	0.111	0.364	0.139
15cpd	0.01±0.02	0.063±0.19	0.036±0.057	0.48	0.051	0.04±0.1	0.063±0.13	0.085±0.129	0.224	0.311	0.156	0.451	0.103
20cpd	0.07±0.14	0.019±0.03	0.022±0.034	0.2	0.052	0.025±0.062	0.04±0.092	0.05±0.078	0.285	0.321	0.164	0.332	0.114
25cpd	0.004±0.01	0.017±0.019	0.014±0.022	0.21	0.048	0.017±0.0044	0.026±0.64	0.029±0.039	0.177	0.405	0.181	0.532	0.128
30cpd	0.0036±0.0086	0.087±0.014	0.01±0.015	0.19	0.06	0.0113±0.03	0.03±0.484	0.019±0.022	0.324	0.422	0.23	0.332	0.159

*cpd* cycles per degree, *MTF* modulation transfer function

*P a*: Comparison of Preoperative and Postoperative 1 Week in PCS Group; *P b*: Comparison of Preoperative and Postoperative 1 Month in PCS Group

*P c*: Comparison of Preoperative and Postoperative 1 Week in FLACS Group; *P d*: Comparison of Preoperative and Postoperative 1 Month in FLACS Group

*P e*: Comparison of PCS Group and FLACS Group at Preoperative; *P f*: Comparison of PCS Group and FLACS Group at Postoperative 1 Week

*P g*: Comparison of PCS Group and FLACS Group at Postoperative 1 Month

Table 3 shows the pre- and postoperative macular thickness values in the FLACS and PCS group. There are not statistically significant after surgery between the two groups. We did not find any difference between pre-operation and post-operation in FLACS group. But in PCS group, there are different between pre-operation and post-operation in nasal inter macular ring thickness (NIMRT) (*p* = 0.03), foveal volume (FV) (*p* = 0.034) and average retinal thickness (ART) (*p* = 0.025) (Table 3, Fig. 1e-f) at 1 month. 
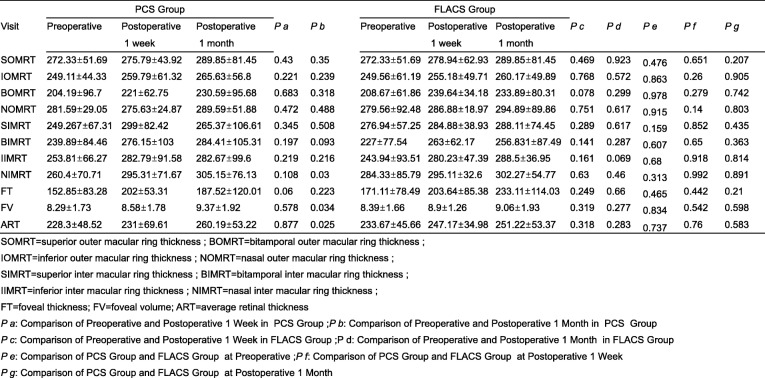

**Table 3:** Comparison of retinal thickness values of PCS group and FLACS group

Visit	PCS Group			*P a*	*P b*	FLACS Group			*P c*	*P d*	*P e*	*P f*	*P g*
	Preoperative	Postoperative	Postoperative			Preoperative	Postoperative	Postoperative					
		1 week	1 month				1 week	1 month					
SOMRT	272.33±51.69	275.79±43.92	289.85±81.45	0.43	0.35	272.33±51.69	278.94±62.93	289.85±81.45	0.469	0.923	0.476	0.651	0.207
IOMRT	249.11±44.33	259.79±61.32	265.63±56.8	0.221	0.239	249.56±61.19	255.18±49.71	260.17±49.89	0.768	0.572	0.863	0.26	0.905
BOMRT	204.19±96.7	221±62.75	230.59±95.68	0.683	0.318	208.67±61.86	239.64±34.18	233.89±80.31	0.078	0.299	0.978	0.279	0.742
NOMRT	281.59±29.05	275.63±24.87	289.59±51.88	0.472	0.488	279.56±92.48	286.88±18.97	294.89±89.86	0.751	0.617	0.915	0.14	0.803
SIMRT	249.267±67.31	299±82.42	265.37±106.61	0.345	0.508	276.94±57.25	284.88±38.93	288.11±74.45	0.289	0.617	0.159	0.852	0.435
BIMRT	239.89±84.46	276.15±103	284.41±105.31	0.197	0.093	227±77.54	263±62.17	256.831±87.49	0.141	0.287	0.607	0.65	0.363
IIMRT	253.81±66.27	282.79±91.58	282.67±99.6	0.219	0.216	243.94±93.51	280.23±47.39	288.5±36.95	0.161	0.069	0.68	0.918	0.814
NIMRT	260.4±70.71	295.31±71.67	305.15±76.13	0.108	0.03	284.33±85.79	295.11±32.6	302.27±54.77	0.63	0.46	0.313	0.992	0.891
FT	152.85±83.28	202±53.31	187.52±120.01	0.06	0.223	171.11±78.49	203.64±85.38	233.11±114.03	0.249	0.66	0.465	0.442	0.21
FV	8.29±1.73	8.58±1.78	9.37±1.92	0.578	0.034	8.39±1.66	8.9±1.26	9.06±1.93	0.319	0.277	0.834	0.542	0.598
ART	228.3±48.52	231±69.61	260.19±53.22	0.877	0.025	233.67±45.66	247.17±34.98	251.22±53.37	0.318	0.283	0.737	0.76	0.583

*SOMRT* superior outer macular ring thickness, *BOMRT* bitamporal outer macular ring thickness, *IOMRT* inferior outer macular ring thickness, *NOMRT* nasal outer macular ring thickness, *SIMRT* superior inter macular ring thickness, *BIMRT* bitamporal inter macular ring thickness, *IIMRT* inferior inter macular ring thickness, *NIMRT* nasal inter macular ring thickness, *FT* foveal thickness, *FV* foveal volume, *ART* average retinal thickness

*P a*: Comparison of Preoperative and Postoperative 1 Week in PCS Group; *P b*: Comparison of Preoperative and Postoperative 1 Month in PCS Group

*P c*: Comparison of Preoperative and Postoperative 1 Week in FLACS Group; *P d*: Comparison of Preoperative and Postoperative 1 Month in FLACS Group

*P e*: Comparison of PCS Group and FLACS Group at Preoperative; *P f*: Comparison of PCS Group and FLACS Group at Postoperative 1 Week

*P g*: Comparison of PCS Group and FLACS Group at Postoperative 1 Month

## Discussion

Optical quality after cataract surgery gained more and more attention for providing satisfactory visual outcomes [20, 21]. Studies have shown that FLACS produce better clear corneal incision morphology [8], more precise reproducible capsulotomies [9–13], and better IOL centration [11] when compared with conventional PCS. Even with these reported benefits, it still needs to be proven whether FLACS can produce better optical quality than conventional PCS. Cataract surgery can cause macular thickness change. The complication has brought substantial attention to surgeons due to its potential hazard to vision consequence [14, 22]. However, the complication changed optical quality still needs to be investigated. Therefore, we studied optical quality by MTF, SR and distinct macular thickness for the two groups in current study.

In our study, the UCDA and CDVA shown no significance between the two groups preoperatively and postoperatively, the results were consisted with previously studies that FLACS did not yield better visual results [4, 23]. Studies have shown that FLACS produce significant reduction in effective phacoemulsification time [23, 24], reduce ultrasound power and ultrasound time [25]. In the research, our results show that FLACS can reduce CDE but not phacoemulsification time. We speculate the reason is that FLACS can pre-chop the lens nuclear. Elevation or rapid fluctuations in IOP may cause vascular or rhegmatogenous events [26]. In FLACS group, we found postoperative IOP were raised, the results are consisted with previous researches [27, 28]. Researches have shown that cytokines in anterior chamber after FLACS are higher than conventional PSC [29]. Therefore, we hypothesize that the raised IOP may be associated with these cytokines which leading to increased resistance at the trabecular meshwork. Whether the raised IOP after surgery affects the retinal nerve fiber layer (RNFL) and visual field which need long-term follow-up and further research.

To better understand optical quality after FLACS, the MTF and SR were measured. MTF is the ratio between the image contrast of a specific object through the imaging optical system and the contrast of the object itself [15]. In general, the higher the MTF and SR, the better the ocular optical quality. In PCS group, we found there are different between pre-operation and post-operation in SR and MTF. Furthermore, the differences are statistically significant when the spatial frequencies are at 5, 10 and 25 cycles/degree (c/d). In FLACS group, we found there are different between pre-operation and post-operation in SR but not MTF. We speculate whether FLACS increase surgery induced astigmatism (SIA) compared with PCS which result in not difference between pre-operation and post-operation for MTF [30]. Even though, MTF value is undoubtedly an objective and accurate indicator for optical quality evaluation [31], the assessment of optical quality cannot be completed by one single indicator. Other objective and subjective indicators should also be integrated to make an accurate assessment. Therefore, we measured DLI in the two groups. The DLI in PCS group is significantly higher than the FLACS group in the baseline measurement (post-operative).

Then, we future analyzed the preoperative and postoperative macular thickness values in PCS and FLACS group by OCT. CME can be detected at the first week and peaks about 4 weeks after surgery [32]. In our study, macular thicknesses were performed before surgery and post-operation at 1 month. In PCS group, there are different between pre-operation and post-operation in NIMRT, FV and ART, but not in FLACS group. The results are consisted with previous research that the FLACS does not difference in postoperative macular thickness as compared with PCS [32].

In this study, the limitation includes a relatively small number of patients and short follow-up period. The other limitation is that the eyes were not randomized for FLACS group or PCS group. The current results might have selection bias, although there are no different in nuclear density between two groups. The comparison of optical quality between FLACS group and PCS group needs a long-term follow-up and further research. Such as capsular fibrosis and posterior capsular opacification (PCO) may cause optical quality change in the two groups. Whether the increasing IOP after FLACS has an effect on the RNFL also requires further investigation.

## Conclusions

Our results suggest although FLACS did not result in macular thickness change compare with PCS, it also cannot gain better optical quality recovery at 1 month after surgery. The slightly retinal change may not affect optical quality in PCS group.

## Supplementary information

**Additional file 1: ** Supplementary Table.

## Data Availability

The datasets used during the current study are available from the corresponding author on reasonable request.

## Abbreviations

ART: Average retinal thickness
BIMRT: Bitamporal inter macular ring thickness
BOMRT: Bitamporal outer macular ring thickness
CDVA: Corrected distance visual acuity
CME: Cystoid macular edema
cpd: Cycles per degree
DLI: Dysfunctional lens index
FLACS: Femtosecond laser-assisted cataract surgery
FT: Foveal thickness
FV: Foveal volume
IIMRT: Inferior inter macular ring thickness
IOL: Intraocular lens
MTF: Modulation transfer function
NIMRT: Nasal inter macular ring thickness
NOMRT: Nasal outer macular ring thickness
OCT: Optical coherence tomography
OMRT: Inferior outer macular ring thickness
PCO: Posterior capsular opacification
PCS: Phacoemulsification cataract surgery
PSF: Point spread function
SIMRT: Superior inter macular ring thickness
SOMRT: Superior outer macular ring thickness
UDVA: Uncorrected distance visual acuity
