# Supplementary material for: Comparison of optical quality and distinct macular thickness in femtosecond laser-assisted versus phacoemulsification cataract surgery
Source: BMC Ophthalmol. 2020 Feb 1;20:42. doi: 10.1186/s12886-020-1319-3 (PMC6995244; doi:10.1186/s12886-020-1319-3)
Supplement: Supplementary file 1 — Additional file 1: Supplementary Table. [file 12886_2020_1319_MOESM1_ESM.docx]

Supplemental Table 1.The grade of lens opacity and identification codes of patients in PCS Group and FLACS Group

| PCS Group | | | |  | FLACS Group | | | |
| --- | --- | --- | --- | --- | --- | --- | --- | --- |
|  | Sex | Age Range | LOCSⅢ |  |  | Sex | Age Range | LOCSⅢ |
| No.1 | male | Group2 | NO3C4P3 |  | No.1 | male | Group3 | NO3C3P2 |
| No.2 | male | Group2 | NO2C3P3 |  | No.2 | female | Group3 | NO3C2P2 |
| No.3 | female | Group2 | NO2C3P2 |  | No.3 | female | Group2 | NO2C4P3 |
| No.4 | female | Group2 | NO2C2P4 |  | No.4 | male | Group2 | NO3C4P2 |
| No.5 | female | Group2 | NO2C2P4 |  | No.5 | female | Group2 | NO4C4P3 |
| No.6 | male | Group2 | NO2C3P2 |  | No.6 | female | Group2 | NO3C2P2 |
| No.7 | female | Group2 | NO3C3P2 |  | No.7 | male | Group2 | NO2C4P2 |
| No.8 | male | Group2 | NO2C2P4 |  | No.8 | female | Group2 | NO3C3P3 |
| No.9 | female | Group2 | NO3C3P3 |  | No.9 | male | Group3 | NO2C4P3 |
| No.10 | male | Group2 | NO3C3P4 |  | No.10 | female | Group2 | NO3C2P2 |
| No.11 | female | Group2 | NO2C2P3 |  | No.11 | female | Group2 | NO2C4P3 |
| No.12 | female | Group1 | NO3C4P2 |  | No.12 | female | Group4 | NO3C3P2 |
| No.13 | male | Group2 | NO3C3P3 |  | No.13 | male | Group2 | NO3C3P3 |
| No.14 | female | Group3 | NO4C4P3 |  | No.14 | female | Group4 | NO3C2P3 |
| No.15 | female | Group2 | NO3C2P2 |  | No.15 | male | Group3 | NO4C4P3 |
| No.16 | male | Group2 | NO3C3P3 |  | No.16 | male | Group3 | NO3C2P2 |
| No.17 | female | Group1 | NO2C4P2 |  | No.17 | female | Group4 | NO2C4P2 |
| No.18 | male | Group2 | NO2C3P3 |  | No.18 | female | Group3 | NO3C2P2 |
| No.19 | female | Group2 | NO3C3P3 |  | No.19 | male | Group2 | NO3C2P2 |
| No.20 | female | Group2 | NO3C2P3 |  | No.20 | male | Group1 | NO2C4P2 |
| No.21 | male | Group2 | NO2C4P2 |  | No.21 | female | Group2 | NO3C2P2 |
| No.22 | female | Group2 | NO2C2P2 |  | No.22 | male | Group3 | NO2C3P2 |
| No.23 | female | Group1 | NO3C3P2 |  | No.23 | female | Group3 | NO2C3P3 |
| No.24 | female | Group2 | NO3C3P2 |  | No.24 | female | Group3 | NO3C3P2 |
| No.25 | male | Group2 | NO3C3P3 |  | No.25 | male | Group3 | NO3C3P3 |
| No.26 | male | Group2 | NO2C4P3 |  | No.26 | male | Group2 | NO3C2P2 |
| No.27 | female | Group1 | NO2C3P3 |  | No.27 | male | Group3 | NO3C2P3 |
| No.28 | male | Group2 | NO3C2P2 |  | No.28 | female | Group2 | NO4C3P2 |
| No.29 | male | Group2 | NO3C3P2 |  | No.29 | male | Group2 | NO2C2P2 |
| No.30 | male | Group2 | NO3C3P4 |  | No.30 | female | Group2 | NO3C2P2 |
| No.31 | female | Group3 | NO3C2P3 |  | No.31 | female | Group3 | NO3C4P3 |
| No.32 | female | Group2 | NO3C2P2 |  | No.32 | female | Group2 | NO3C4P2 |
| No.33 | male | Group2 | NO3C3P3 |  | No.33 | male | Group3 | NO3C3P3 |
| No.34 | female | Group2 | NO2C4P3 |  | No.34 | female | Group3 | NO2C2P3 |
| No.35 | female | Group2 | NO3C4P2 |  | No.35 | male | Group3 | NO2C4P3 |
| No.36 | male | Group3 | NO2C3P3 |  | No.36 | male | Group2 | NO3C2P2 |
| No.37 | female | Group2 | NO3C4P2 |  | No.37 | female | Group3 | NO2C2P4 |
| No.38 | male | Group2 | NO2C3P3 |  | No.38 | female | Group2 | NO3C2P2 |
| No.39 | female | Group3 | NO3C3P3 |  | No.39 | male | Group3 | NO3C2P3 |
| No.40 | female | Group1 | NO3C2P3 |  | No.40 | male | Group3 | NO2C4P2 |
| No.41 | male | Group2 | NO3C3P2 |  | No.41 | female | Group3 | NO2C2P2 |
| No.42 | female | Group3 | NO3C2P2 |  | No.42 | male | Group3 | NO2C2P4 |
| No.43 | female | Group3 | NO3C3P2 |  | No.43 | female | Group1 | NO2C3P3 |
| No.44 | female | Group1 | NO3C2P2 |  | No.44 | female | Group3 | NO3C2P2 |
| No.45 | male | Group3 | NO2C3P4 |  | No.45 | male | Group2 | NO2C3P3 |
| No.46 | male | Group1 | NO2C2P4 |  | No.46 | male | Group3 | NO4C2P2 |
| No.47 | female | Group2 | NO2C3P3 |  | No.47 | male | Group3 | NO2C2P3 |
| No.48 | male | Group3 | NO3C3P2 |  | No.48 | female | Group3 | NO3C3P3 |
| No.49 | male | Group3 | NO3C4P2 |  | No.49 | male | Group3 | NO2C4P2 |
| No.50 | male | Group3 | NO3C2P4 |  | No.50 | female | Group3 | NO3C3P3 |

Group1:41-50y, Group2:51-60y, Group3:61-70y, Group4:71-80y
